# Structural insights on the nucleoprotein C-terminal domain of Měnglà virus

**DOI:** 10.1128/spectrum.02373-23

**Published:** 2023-10-27

**Authors:** Diego Sebastian Ferrero, Omar Tomás Gilabert, Nuria Verdaguer

**Affiliations:** 1 Molecular Biology Institute of Barcelona (IBMB-CSIC), Barcelona, Spain; University of Arizona, Tucson, Arizona, USA

**Keywords:** filovirus, inclusion body, Měnglà virus, nucleoprotein

## Abstract

Filoviruses depend on the nucleoprotein (NP) to accomplish multiple functions during the viral life cycle. NP is the most abundantly expressed viral protein in infected cells and the main component of the viral nucleocapsid. It can be structurally divided into amino- and carboxy- terminal domains (NTD and CTD). The NTD can homo-oligomerize to interact and protect the (−) ssRNA genome, forming long helical structures. The flexible CTD is responsible for the binding of other nucleocapsid proteins and is involved in the formation of inclusion bodies (IBs)—the cytoplasmic sites of nucleocapsid formation and genome replication. The CTD ends in a ~100-residue globular tail. Měnglà virus (MLAV) is the only member of the new *Dianlovirus* genus within the *Filoviridae* family. Their differential characteristics and the possibility of becoming a threat for human health justify the interest in better understanding of its structure and function. In this work, we present the structure of the globular tail of the MLAV NP CTD, showing an overall conformation closely related to that previously reported for the equivalent NP region in MARV. Moreover, analyses of the CTD-CTD interactions in the crystal asymmetric unit revealed a higher-order helicoidal structure. Mutational studies underscore the crucial role of a number of residues, located at the CTD-CTD contact interface, for IB formation. Site-directed mutagenesis of amino acids L653 and F687, involved in the formation of these helicoidal assemblies, abrogate the growth of IBs when the full-length MLAV NP was ectopically expressed in cells. Our findings confirm the role of NP CTD in IB formation, also for MLAV, and focus the attention on particular residues as starting point for further analysis.

**IMPORTANCE:**

Filoviruses are the causative agents of severe and often fatal hemorrhagic disease in humans. Měnglà virus (MLAV) is a recently reported filovirus, isolated from fruit bats that is capable to replicate in human cells, representing a potential risk for human health. An in-depth structural and functional knowledge of MLAV proteins is an essential step for antiviral research on this virus that can also be extended to other emerging filoviruses. In this study, we determined the first crystal structures of the C-terminal domain (CTD) of the MLAV nucleoprotein (NP), showing important similarities to the equivalent domain in MARV. The structural data also show that the NP CTD has the ability to form large helical oligomers that may participate in the control of cytoplasmic inclusion body formation during viral replication.

## INTRODUCTION

Filoviruses are enveloped, non-segmented negative-sense RNA viruses with filamentous morphology. The *Filoviridae* family includes important pathogenic members capable of causing severe hemorrhagic fever diseases in humans and non-human primates with high lethality. Ebola (EBOV) and Marburg (MARV) viruses are the best-known examples (1). The natural wildlife host of EBOV has not been definitively identified but specific bat species may represent a reservoir (1 – 5). The MARV reservoir is found in Egyptian fruit bats (*Rousettus aegyptiacus*) (6). The unpredictable re-emergence of filoviruses and the discovery of diverse variants with potential risk for the human health (7, 8) exacerbate the threat of future outbreaks.

Měnglà virus (MLAV) is a recently characterized filovirus isolated from *Rousettus* bats that is capable to replicate in human cells, representing a potential risk for human health (8). MLAV has a general genome organization identical to other filoviruses but containing enough differences to be considered a member of a new genus, Dianlovirus (8). Like other Filoviruses, the ~19 kb RNA MLAV genome encodes seven proteins: the nucleoprotein (NP), the surface glycoprotein (GP), the viral proteins VP35, VP40, VP24, VP30, and the RNA-dependent RNA polymerase (L). NP is abundantly expressed in infected cells and directly binds and envelops the viral genome, being essential for the virus replication cycle as the main component of the viral nucleocapsid (NC). Besides NP, NC contains VP35, VP24, VP30, and L.

NP is a ~70 kDa protein with ~40% global sequence homology between EBOV, MARV, and MLAV counterparts (8 – 10). NP can be divided into a hydrophobic N-terminal domain (~400 residues) and a hydrophilic C-terminal moiety (~300 residues). The N-terminal domain is responsible for direct RNA binding and NP-NP interactions (10). The NP C-terminal domain (CTD), formed by a long intrinsically disordered linker and a structured tail subdomain, is the most variable part of the protein. It protrudes from the helical NC in virus-like particles (11, 12) and is responsible for the interaction with the matrix protein VP40, the incorporation of NP and the RNA genome into virions (13, 14), and the formation of inclusion bodies (IBs) (15, 16). IBs are dynamic cytoplasmic proteinaceous structures observed in Filoviruses and other negative-sense RNA viruses (17 – 22) housing RNA synthesis. In addition to the protection of the viral RNA from cellular defenses, filoviral IBs contain the host and viral proteins (VP30, VP35, VP24, and L) required for viral RNA replication and virus maturation (15, 16, 19). It has also been shown that the presence of NP and in particular, the NP CTD is necessary for IB formation (15).

Given the critical role of NP for the viral life cycle, many efforts to control the filovirus infection are directed at the production of antibodies against multiple NP epitopes (23 – 25) or drugs targeting the NP domains (26). Hence, the availability of structural information is critical not only to understand the NP function but also to design more efficient therapies (27).

The structure of both NP domains from different filoviruses has been previously determined by X-ray crystallography or cryo-EM (28 – 33). However, NP CTD appeared to be more difficult to crystallize due to its flexibility (34 – 36). An extreme situation was MARV NP CTD, a close relative of MLAV, which only crystallized in complex with nanobodies that stabilized its structure (37).

In this work, we determined the crystal structures of MLAV NP CTD in three different space groups (P1, P2_1_, and P3_2_), showing important similarities to the equivalent domain in MARV. These structures also revealed a conserved helical organization of this domain in the packing of the three crystal forms analyzed. Interestingly, some amino acids involved in the contact interface that support this helical arrangement appear to be relevant for IB formation, when the full-length NP is transiently expressed in cells. This suggests a possible link between this particular quaternary organization of the MLAV NP CTD and the formation of IBs.

## RESULTS

### Characterization of protein constructs

**Fig 1:**
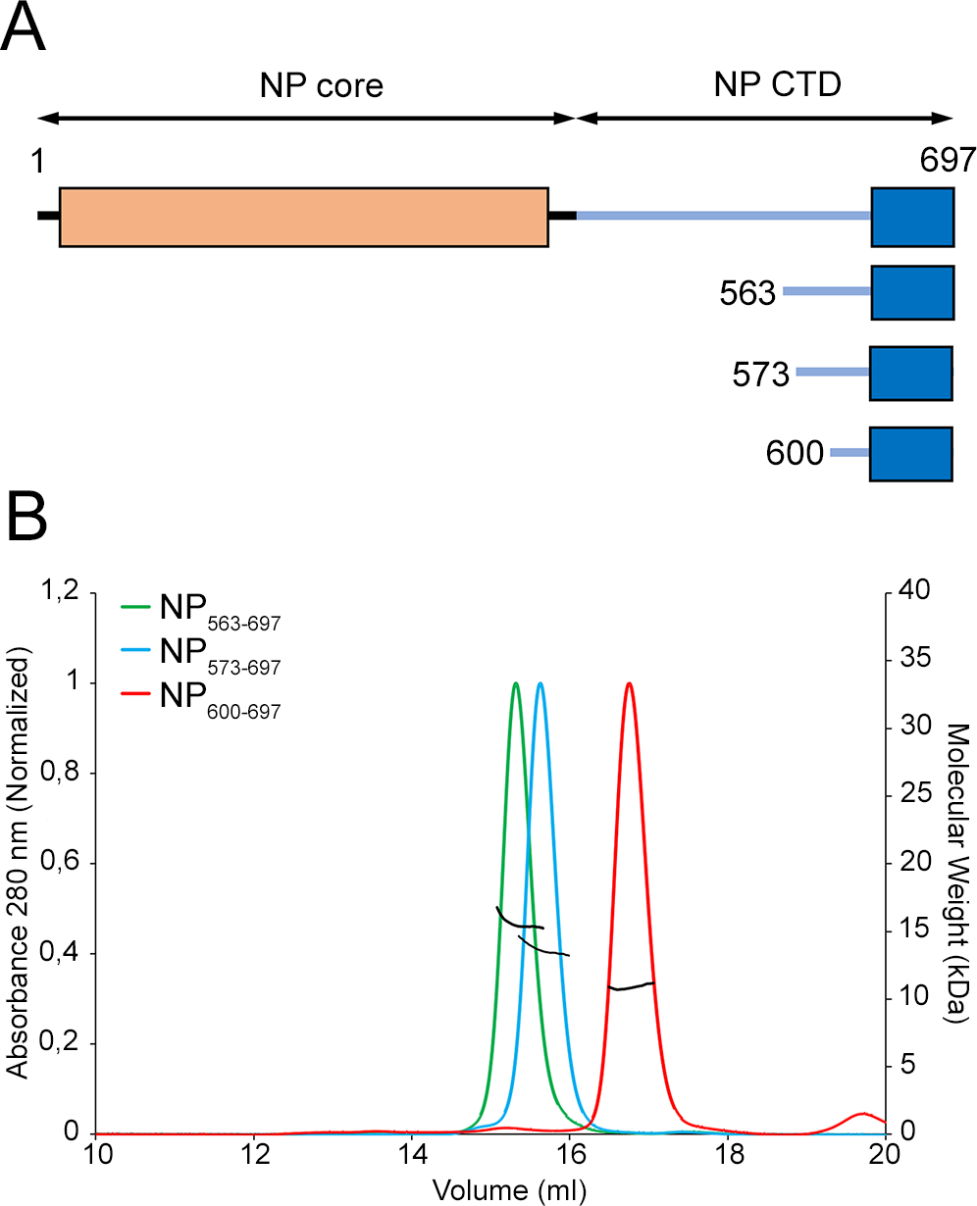
The C-terminal domain of MLAV NP. (**A**) Schematic diagram of MLAV NP protein (residues 1–697) and recombinant protein constructs (563–697, 573–697, and 600–697) expressed as GST C-terminal fusion protein. (**B**) SEC-MALS profiles of MLAV NP constructs after GST digestion and removal, in buffer 50 mM Tris pH 8.0, 500 mM NaCl, 5 mM DTT, 5% (vol/vol) glycerol. The black lines intercepting each pick correspond to the measured molecular masses shown in the right axis (563–697: 15.64 ± 0.03 kDa; 573–697: 13.67 ± 0.06 kDa; and 600–697: 10.87 ± 0.03 kDa. The theoretical MWs for each construct monomer are 15.7, 13.9, and 11.0 kDa, respectively.

In order to obtain a soluble and crystallizable construct of MLAV CTD, three constructs of this domain were generated (Fig. 1A) and expressed as GST fusion proteins. All of them were soluble even after GST removal, yielding NP_563–697_, NP_573–697_, and NP_600–697_ proteins. To verify the structural integrity and correct folding, the cleaved proteins were subjected to size exclusion chromatography (SEC) coupled to multiangle light scattering (MALS), revealing that all three samples were monodisperse with molecular weights (MWs) of 15.64 ± 0.03, 13.67 ± 0.06, and 10.87 ± 0.03 kDa, respectively. These values perfectly matched with the theoretical MWs for monomers of each construct (15.6, 13.9, and 14.67 kDa, respectively). The purified proteins were subjected to multiple crystallization trials and the constructs NP_573–697_ and NP_600–697_ yielded single crystals in different conditions (see M&M section). However, only NP_573–697_ crystals diffracted at reasonable resolution, allowing the structure determination (Table 1).

**TABLE 1:** Data collection and refinement statistics^*a*^

PDB ID	8P10	8P24	8P0Y
Data collection			
Beamline (Synchrotron)	I03 (DIAMOND)	XALOC (ALBA)	XALOC (ALBA)
Wavelength (Å)	0.9762	0.9792	0.9793
Resolution range (Å)	142.16–3.26 (4.05-3.26)	173.13–3.73 (4.27-3.73)	48.90–4.12 (4.44-4.12)
Lowest cutoff iffraction limit (Å) (direction)^*b*^	4.46 (−0.223a*+0.949b*+0.223c*)	8.18 (−0.498a*+0.830b*+0.249c*)	7.57c*
Best diffraction limit after cutoff (Å) (direction)^*b*^	3.25 (0.365a*+0.097b*+0.926c*)	3.73 (0.044a*+0.022b*+0.999c*)	4.12 (0.993a*+0.114b*−0.028c*)
Space group	P1	P2_1_	P3_2_
Unit cell parameters			
a, b, c (Å)	68.31, 85.71, 146.24	56.75, 162.94, 175.36	177.18, 177.18, 90.95
α, β, γ (°)	88.5, 76.4, 80.3	90.0, 99.2, 90.0	90.0, 90.0, 120.0
Measured reflections	113,521 (22,779)	30,131 (1,356)	61,985 (3,272)
Unique reflections	30,881 (6,177)	8,269 (414)	12,572 (660)
R_merge_ ^*c*^	0.103 (0.713)	0.131 (0.787)	0.284 (1.821)
Rpim^*f*^	0.063 (0.431)	0.079 (0.494)	0.143 (0.917)
Multiplicity	3.7 (3.7)	3.6 (3.3)	5.0 (4.9)
Completeness (%)			
Spherical	62.1 (25.1)	25.2 (33.8)	51.1 (11.8)
Ellipsoidal	89.2 (76.9)	84.1 (55.1)	83.8 (46.9)
Mean I/Sigma(I)	8.2 (1.9)	4.7 (1.8)	5.4 (1.6)
Half-set correlation CC (1/2)	0.998 (0.710)	0.995 (0.785)	0.989 (0.449)
Refinement			
R_work_ ^*d*^	0.267	0.248	0.235
R_free_ ^*e*^	0.297	0.265	0.264
Protein residues	1,833	1,804	989
RMS (bonds)	0.004	0.030	0.005
RMS (angles)	0.722	0.670	0.820
Ramachandran plot (%)			
Favored	93.18	93.44	92.55
Allowed	6.65	6.41	7.45
Clash score	11.64	15.90	14.59

^*a*^ The number in parentheses refers to the last (highest) resolution shell.

^*b*^ Data were processed using anisotropic resolution limits using STARANISO server.

^*c*^ Rmerge = Σ|I j − <I>| / Σ I j where I j is the intensity of an individual reflection and <I> is the average intensity of that reflection.

^*d*^ Rwork = Σhkl ||Fobs(hkl)|—|Fcalc(hkl)|| / Σhkl |Fobs(hkl)|, where Fobs and Fcalc are the structure factors, deduced from measured intensities and calculated from the model, respectively.

^*e*^ Rfree = as for Rwork but for 5% of the total reflections chosen at random and omitted from refinement.

^*f*^ Rpim = ∑ hkl √1/n−1∑∣I_i_ (hkl)−̄I (hkl)∣ / ∑ ∑ I_i_ (hkl).

### Overall structure and quaternary arrangement of the NP CTD

**Fig 2:**
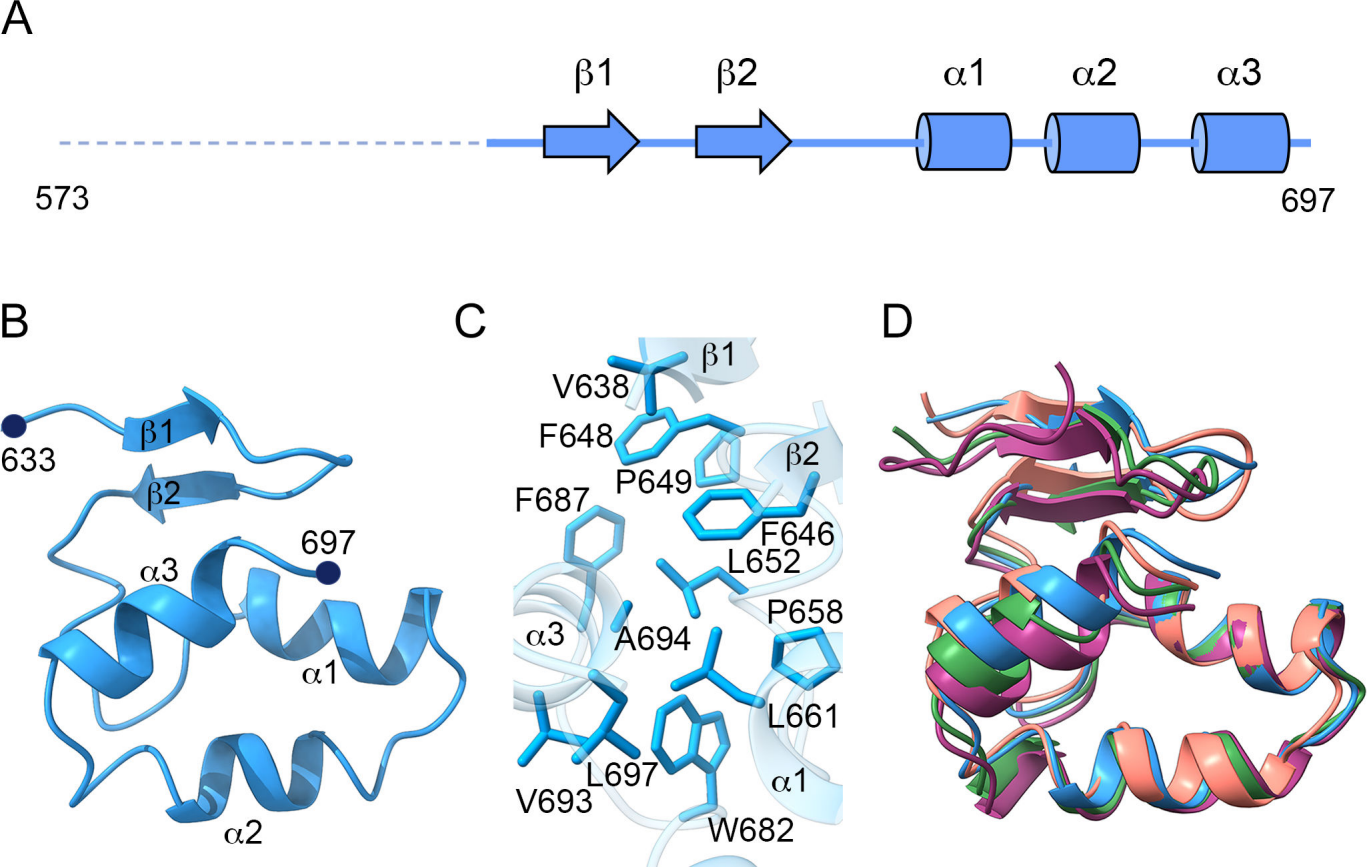
Structure of the C-terminal domain of MLAV NP. (**A**) The secondary-structural elements of the MLAV NP C-terminus. The α-helices are shown in blue cylinders and the β-strands as blue arrows. (**B**) Ribbon diagram, showing the structure of the MLAV C-terminal tail, as seen in the P1 crystals, with the secondary structural elements explicitly labeled. The visible structure corresponded to residues from D633 to L697. (**C**) Hydrophobic residues in the contact interfaces of α1, α2, α3, the coil region, and the β-hairpin. (**D**) Structural superimposition of four representative MLAV NP C-terminal tail structures of the 28 molecules present in the asymmetric unit of the P1 crystals.

We have determined the structure of MLAV CTD (NP_573–697_) in triclinic (P1), monoclinic (P2_1_), and trigonal (P3_2_) crystal forms (Table 1), containing 28, 28, and 14 CTD molecules in the crystal asymmetric units (AUs), respectively. The structures were solved by molecular replacement (MR). The P1 crystals were solved first, using the coordinates of the closely related NP CTD domain of MARV (PDB: 4W2Q, residues 633–695, harboring 42% sequence identity) (37). Initial MR solutions were manually edited to build the correct sequence of the MLAV polypeptide in the electron density maps. The visible structure corresponded to the tail subdomain, spanning residues from approximately Q634 to the NP C-terminus (L697; Fig. 2A and B). The amino acids visible in the N-terminal region vary from one molecule to another in the AU, allowing the main chain tracing from Q631 in some of the molecules. The most N-terminal part of the construct, belonging to the predicted intrinsically disordered linker (residues 573–630), was not visible in any of the chains. Hence, the structure determined comprised an initial β-hairpin, formed by the antiparallel strands β1 (636–639) and β2 (645–649), a coil fragment with a short helical region and three C-terminal helices α1 (659–668), α2 (672–684), and α3 (687–695) (Fig. 2A and B). The three helices contacted with each other, burying most of their hydrophobic residues (P658, L661, A664, L665, L687, W682, V693, A694, and L697) in the interactions. The small hydrophobic patch formed by α1 and α3 were in contact with other hydrophobic residues in the coil region (L652) and in the β-hairpin (V638, F646, F648, and P649) (Fig. 2C).

**Fig 3:**
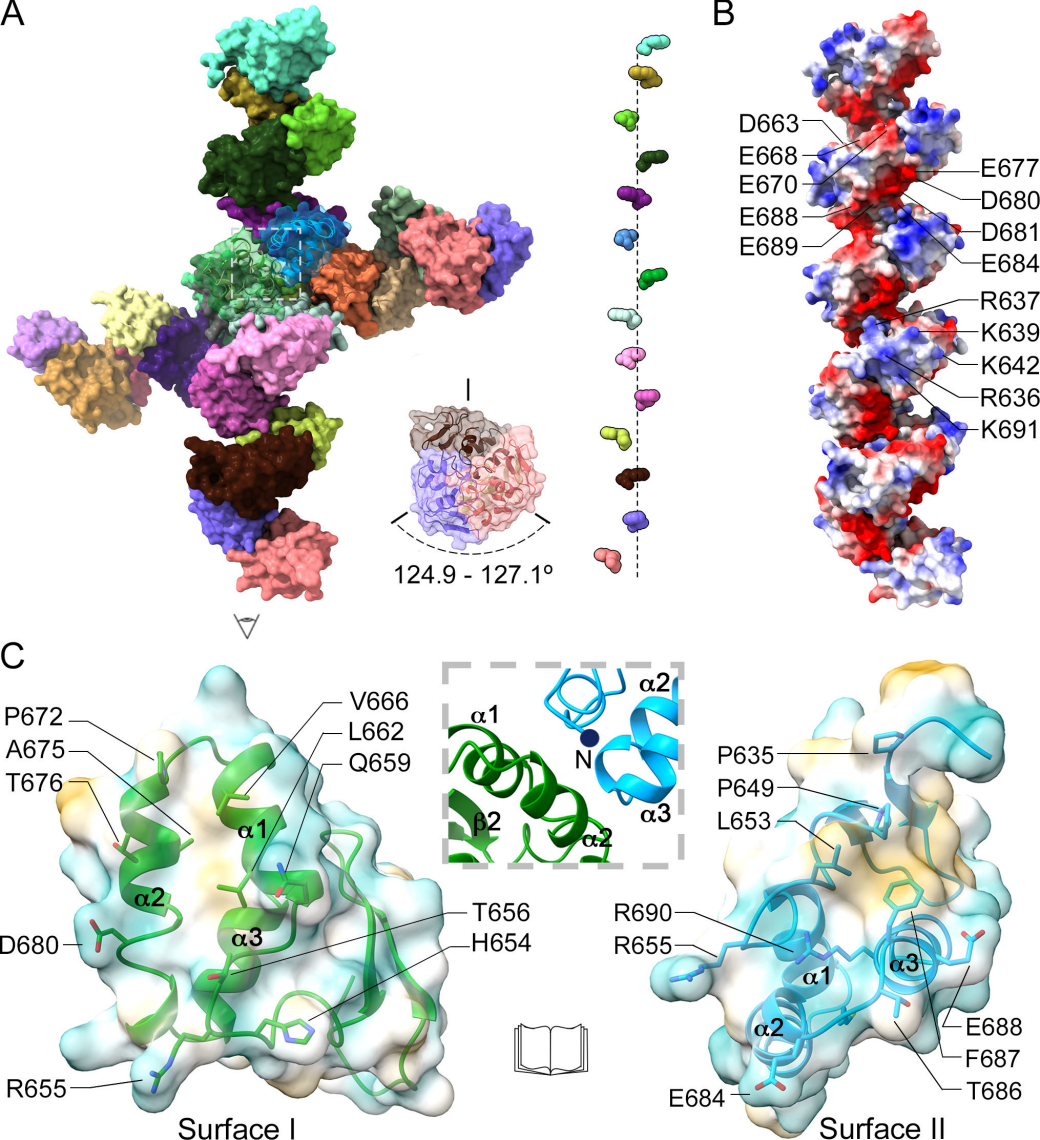
Helicoidal arrangement of the MLAV NP C-terminal tail. (**A**) The quaternary arrangement of MLAV NP CTD in the P1 crystals. The 28 molecules of the crystal asymmetric unit are highlighted in different colors. The middle insets show the relative disposition of the domain monomers in one helical turn and along the pseudo-threefold axis. The domain protomers were located along a pseudo-threefold axis describing a variable rotation, ranging from 124.9° to 127.1°, between one molecule and its neighbor. Representation of W682 as a marker on each monomer along one axis to show the misaligned helicoidal arrangement. (**B**) Surface representation of one MLAV NP CTD helix with the electrostatic potential colored in blue and red for positive and negative charges, respectively. It is worth noting the regular arrangement of the electropositive and electronegative patches along the length of the helix. (**C**) Conserved MLAV NP CTD self-interactions stabilizing the formation of the long helices. Molecules I (green) and II (cyan) are shown as an open book representation of the region boxed in (A) and their hydrophilic surfaces are colored dark-cyan and hydrophobic regions are colored dark-goldenrod. The interface of interaction involves residues of helices α1 and α2, and strand β2 of one molecule (surface I), and the N-terminus and helices α2 and α3 of the second molecule (surface II). The side chains of the interacting amino acids in the interface are shown as sticks and explicitly labeled. The inset between the two molecules is a closeup representation of the contact region in the original orientation.

The Cα superposition of the 28 independent copies of the NP CTD tail in the P1 crystals showed an almost identical fold, with averaged r.m.s.d. values of 0.5 ± 0.3 Å. The highest flexibility was observed in the C-terminal helix α3 and in the relative position of the N-terminal β-strands, with respect to α1 and α2 (Fig. 2D). These 28 independent molecules were organized in two striking linear dextro-helical oligomers (14 molecules each; Fig. 3A and B), forming an angle of ~107° between them (Fig. 3A). The protomers were located along a pseudo-threefold screw axis, describing a variable rotation ranging from 124.9° to 127.1° between one molecule and its neighbor (Fig. 3A inset). The visible N-terminal ends of each protomer were oriented toward the large solvent cavity, as if the flexible linker region protruded from the structure. This helical arrangement was maintained by repetitive contacts of the protomers, involving an interface of 590 ± 44 Å^2^ on average (~14% of the domain surface). This buried interface involved polar and hydrophobic interactions, mediated by residues of helices α1, α2, and the strand β2 of the first molecule (surface I) that are in contact with residues of α2 and α3 from the neighboring molecule (surface II) (Fig. 3C). The surface I residues D680, R655, T656, and H654 were involved in polar interactions with amino acids R655, D684, T686, E688, and R690 of surface II (Fig. 3C). In addition, the surface I residues L662, V666, P672, and A675 formed a hydrophobic cavity, where the L653 side chain of the neighboring molecule (surface II) was buried (Fig. 3C). L653 was the most protruding residue of the hydrophobic cluster on surface II, also formed by the amino acids F687 and P649 (Fig. 3C).

**Fig 4:**
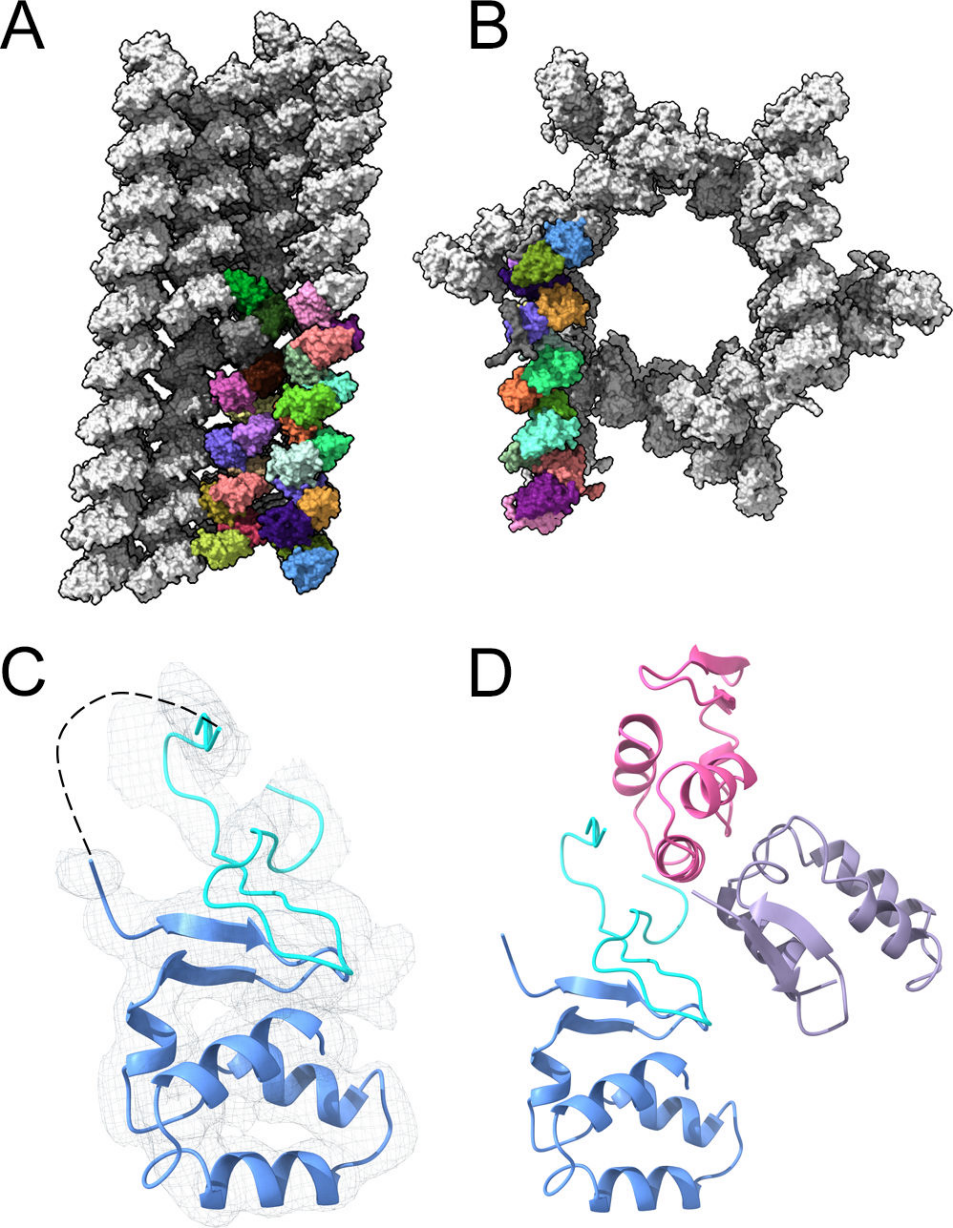
The quaternary arrangement of the MLAV NP CTD in the P2_1_ and P3_2_ crystal forms. (**A**) The molecular packing in the P2_1_ crystals, showing the 28 independent molecules of the asymmetric unit (depicted in different colors), organized in a quasi-parallel orientation. (**B**) The molecular packing in the P3_2_ crystals, showing multiple 14-molecule super-helices (monomers in different colors), stacked along the *z*-axis, describing an hexagonal channel of solvent of ~120 Å diameter. (**C**) Cartoon representation and electron density (gray mesh), corresponding to one of the two NP CTD monomers (blue) with additional density (cyan), which was interpreted as the flexible N-terminal moiety of the NP construct, with unassigned sequence. (**D**) Packing contacts supporting the partially ordered structure of the CTD N-terminal moiety. In this position, the CTD N-terminus interacted with two neighboring molecules (pink and purple cartoons) in the crystal packing, facilitating the visualization of its structure in the electron density maps.

The final refined models of the CTD tail obtained from the P1 crystals were used as MR models to solve both the monoclinic and trigonal structures. The entire coordinates of one of the two 14-molecule super-helices, forming the P1 crystals were used as the initial model to solve the structure of the P2_1_ crystals. Two super-helices were also present in the AU of these crystals, and these oligomers were organized in a quasi-parallel position, describing a ~5° angle and generating a more compact packing than in the P1 space group (Fig. 4A).

In contrast, the AU of the P3_2_ crystals was defined by only one helical oligomer (Fig. 4B). A peculiarity of this structure was the presence of additional continuous densities in close proximity to the N-terminal β-hairpin in some of the CTD molecules of the AU. In one particular case, the electron density maps allowed the main chain tracing of 32 additional residues that would correspond to the N-terminal linker (Fig. 4C and D). It is likely that the proximity of two neighboring molecules in the crystal packing facilitated the folding of this flexible region (Fig. 4D). Unfortunately, the limited resolution prevented from assigning with confidence the correct sequence.

The five super-helices found in the AUs of the different crystals could be easily superimposed. A close inspection of the electrostatic surface along these helical oligomers showed the presence of clusters of both positively and negatively charged residues (Fig. 3B). The negative charges were concentrated in the groove along the screw axis, while small positive clusters were periodically situated on the crest. The electronegative surface was formed by residues E677, D680, D681, E688, E689, and E684 of one monomer and D663, E668, and E670 of the third monomer in the pseudo-threefold screw axis. The positive patch was formed by the N-terminal residues R636, R637, K639, and K642, located in the β-hairpin, and K691 of α3 (Fig. 3B).

### Structural similarities with other filovirus NP tails

**Fig 5:**
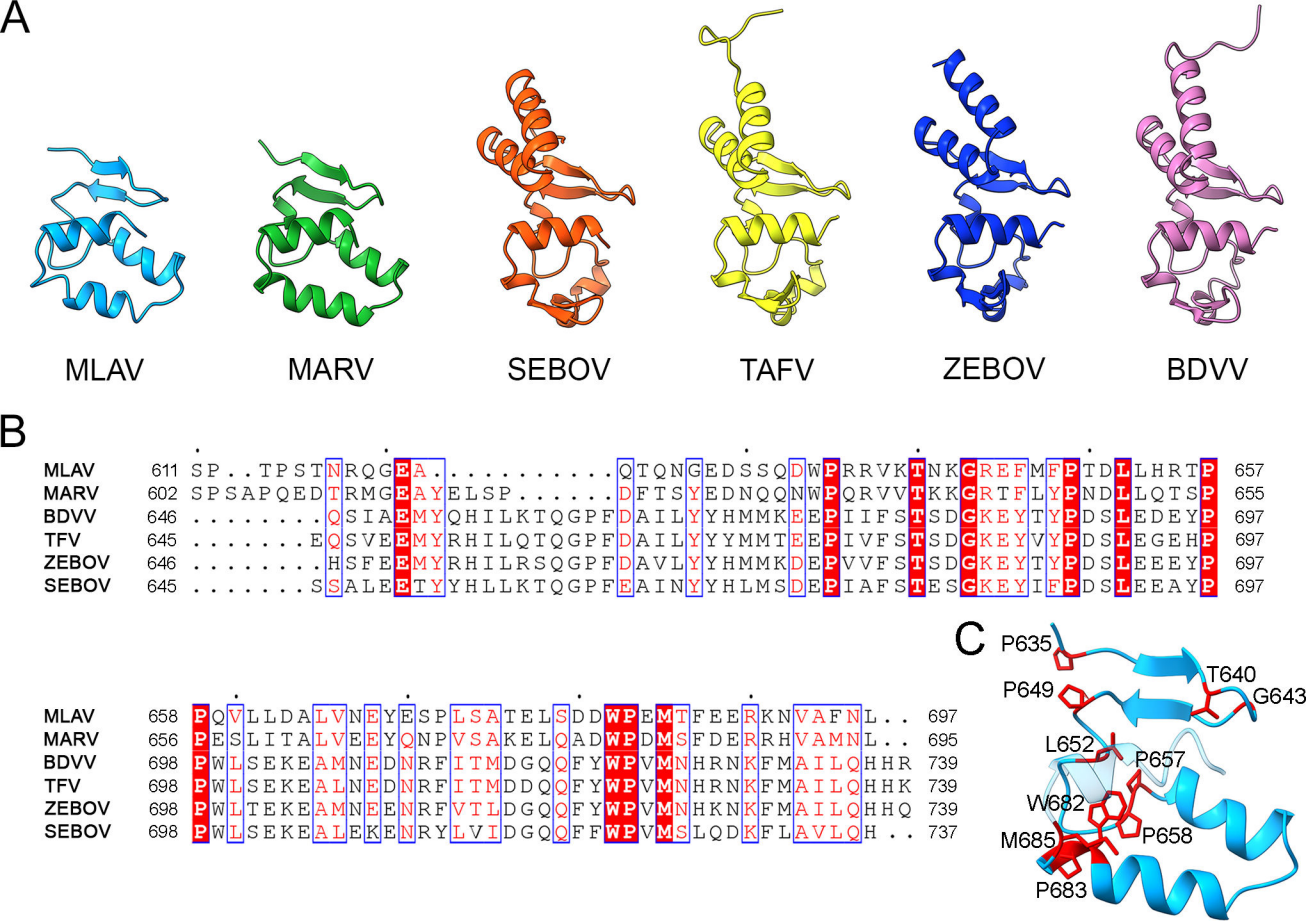
Structural comparisons and sequence alignments of the C-terminal tails of NP proteins of known structure. (**A**) Cartoon representation of the tail structures of MLAV (cyan, this work), MARV (green, PDB:4W2O) (37), Sudan ebolavirus (SEBOV, orange; PDB:6U51) (25), Taï Forest Ebolavirus (TAFV, yellow; PDB:5E2X) (34), Zaire ebolavirus (ZEBO, blue; PDB:4QAZ) (36), and Bundibugyo Ebolavirus (BDVV, pink; PDB:5DSD) (38). (**B**) Sequence alignments corresponding to the PDB structures mentioned above. The strictly conserved residues are in red blocks and similar residues are in red characters. Upper points represent intervals of 10 protein residues. (**C**) Cartoon representation of the MLAV CTD showing the positions of the strictly conserved residues among filovirus.

The NP CTD is less conserved than the NP NTD among filoviruses (36). Structure and sequence alignments of the ~95 most C-terminal residues from different filoviruses revealed a variable structure and generally low sequence identity, ranging from ~17% to 46% (Fig. 5A and B), being the MARV tail the most similar to MLAV. Only 11 residues are strictly conserved among the different viruses and most of them are located on one side of the structure in the convergence of the secondary structural elements of the domain (Fig. 5C).

Despite the low homology in the NP CTD sequences among the different filoviruses (Fig. 5B), the MLAV and MARV NP CTD (PDB: 4W2O; 37) structures can be easily superimposed with a r.m.s.d. of 1.0 Å (for the superimposition of 63 residues). In contrast, the partial superimposition of the MLAV NP CTD coordinates (30 residues only), with the equivalent domains in the different EBOV strains [SUDAN (PDB:6U51; 39), TAFV (PDB: 5E2X; 34), ZEBO (PDB:4QAZ; 36), and BDVV (PDB: 5DSD, 38)] exhibit higher r.m.s.d values (from 1.53 to 1.76 Å). The CTD structures of MLAV and MARV lack the N-terminal α-helical hairpin of the EBOV strains that is apparently replaced by an unstructured region. However, we have been able to define a structure for this region in one of the CTD molecules of the AU in the trigonal crystals. In this position, the N-terminal region of this domain appeared tightly packed with other molecules in the crystal packing (Fig. 4C and D).

### Small angle X-ray scattering data confirm the flexible nature of the NP CTD linker region preceding the globular C-terminal tail

**Fig 6:**
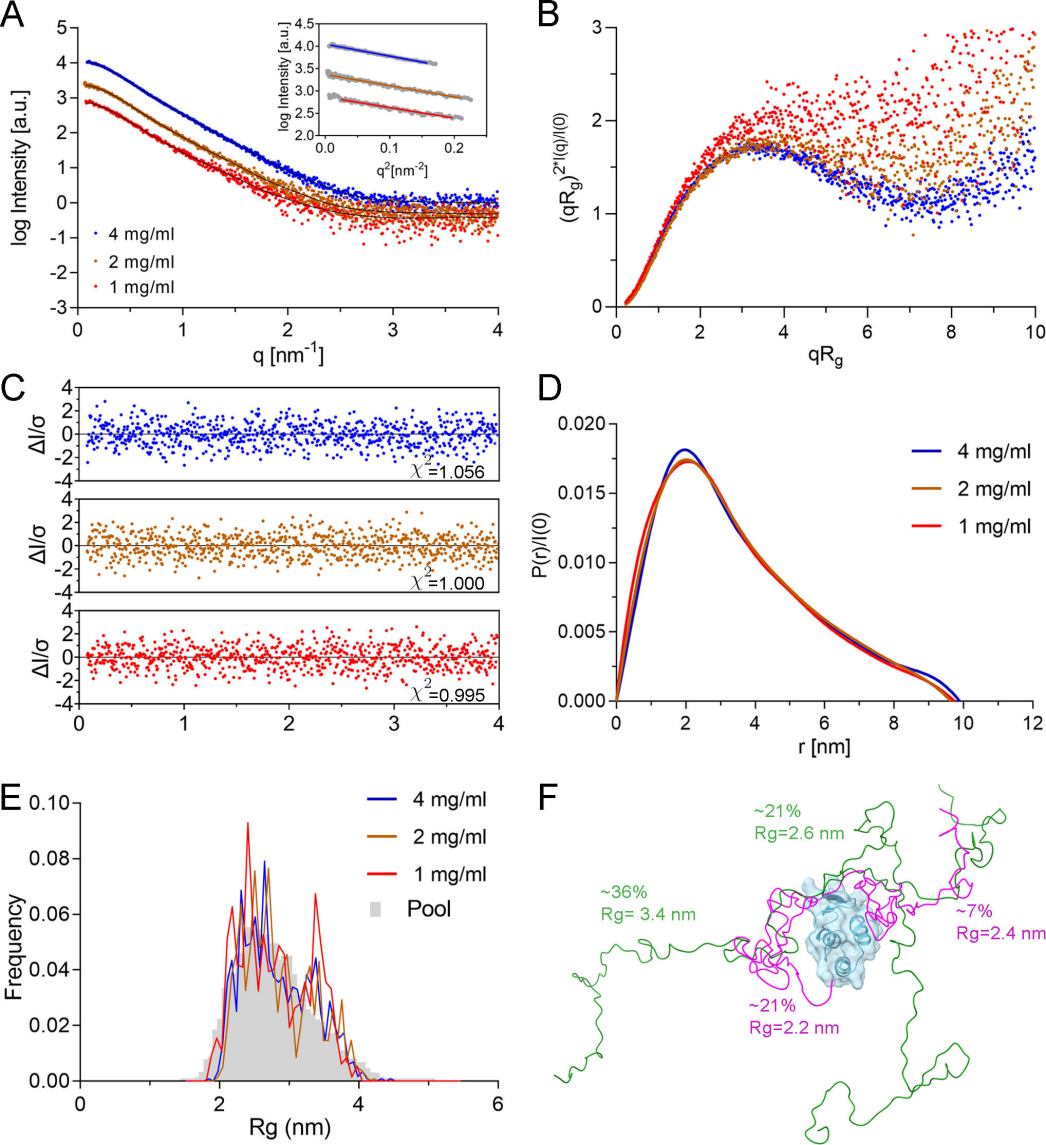
SAXS data of NP_573–697_ protein construct. (**A**) Solution scattering data collected for the NP_573–697_ CTD construct at different concentrations (blue 4 mg/mL; brown 2 mg/mL; red 1 mg/mL). EOM fits to the experimental data are displayed as black lines. The inset shows the Guinier plot and data fitting for Rg calculation (see Table 2). (**B**) Dimensionless Kratky plot of the different protein concentrations, using the same color code as in panel (A). (**C**) Plots of the error-weighted residuals of the EOM modeling fitting for each protein concentration curve [color code as in panel (A)]. χ2 values for fitting are shown on each graph. (**D**) Comparison of the pair distance distribution function *P*(*r*) for all data. (**E**) Rg distributions for EOM fits. The random starting pools (gray bars) and selected ensembles for NP_573–697_ at different protein concentrations (color lines). (**F**) Models of NP_573–697_ obtained from EOM modelling. The rigid model, corresponding to the crystal structure solved in this work, is depicted in cartoons (cyan) and surface representation. The modeled flexible tails are shown in green and magenta for extended and compact conformations, respectively. Numbers indicate the frequency in the selected pool for each model.

To obtain additional information about the structure of the crystallized NP_573–697_ construct in solution, we collected small angle X-ray scattering (SAXS) data at three different concentrations (1–4 mg/mL; from 71.9 to 287.8 µM) (Fig. 6A through C) and calculated the MW derived from this data, using the Volume of correlation (Vc) method (40). The calculated MW of the construct at each protein concentration was slightly similar to the theoretical MW of the monomeric protein (13.9 kDa), indicating that NP_573–697_ at low protein concentration is a monomer in solution, in agreement with the SEC-MALS data (Fig. 1B). Two additional structural parameters were calculated from the SAXS curves, the radius of gyration (Rg) and the maximum diameter of the particle (Dmax) (Table 2). Rg indicates the average distance of all the atoms in the protein from its center of mass and describes the overall size of the macromolecule. Therefore, the Rg value could be used to monitor the conformational changes or the oligomerization state of the macromolecule. The Rg values determined for the NP_573–697_ construct are 2.73–2.93 nm, rather large for a protein of this MW. For comparison, bovine serum albumin has 519 amino acids (MW: 66.4  kDa,) and an Rg = 2.8  nm (41). Taking into account the monomeric state of this protein construct, the observed Rg values would indicate an elongated shape of the NP_573–697_ in solution.

**TABLE 2:** SAXS parameters

NP_573–697_	Rg [nm]	Dmax [nm]	Dmax/Rg	I(0)	MWvc [kDa]
1 mg/mL	2.78 ± 0.02	9.75	3.51	17.4 + 0.11	12.48
2 mg/mL	2.73 ± 0.02	9.64	3.53	29.0 ± 0.13	13.88
4 mg/mL	2.82 ± 0.02	9.89	3.50	57.1 ± 0.13	15.72

On the other hand, Dmax is obtained from the distance distribution function [*P*(*r*)] that is a histogram of distances in real space between all possible pairs of atoms within a particle. This function can be obtained from experimental scattering data using indirect Fourier transformation (42). In addition, some structural properties can be obtained from *P*(*r*), e.g., globular and compact proteins have a symmetric and bell-shaped function but not unfolded proteins (42). The *P*(*r*) for all the analyzed NP_573–697_ concentrations are asymmetric and tailed off to large distances. This is compatible with the profile of extended proteins (Fig. 6D).

The Dmax/Rg ratio is also an indicator of the shape anisotropy of the particle and usually is within the range of 2  <  Dmax/Rg  <  4, where compact proteins have Dmax/Rg  =  2, globular proteins have Dmax/Rg  =  3 and extended proteins Dmax/Rg  =  4. The calculated Dmax/Rg ratio values for NP_573–697_ protein are presented in Table 2. The measured Dmax/Rg ratio values are well above 3, indicating an elongated shape of the protein construct.

The dimensionless Kratky plots that qualitatively analyze the protein flexibility and disorder were similar for all protein concentrations, showing the typical shape with nonapparent maximum, characteristic of proteins containing mixed globular/disordered parts (Fig. 6B).

Based on the described SAXS parameters, we calculated models of the monomeric NP_573–697_ structure in solution using the Ensemble Optimization Method (EOM), which try to describe the experimental SAXS data using an ensemble of atomic models (43). Using one crystal structure obtained in this work as a model and assuming the remaining residues in NP_573–697_ arranged as a flexible random chain, EOM found solutions with a very good fit with experimental data, with *χ*2 values of 0.995, 1.000, and 1.056 for the 1, 2, and 4 mg/mL curves, respectively (Fig. 6A and C).

The analysis of the Rg distribution for the selected EOM ensembles of models shows that in comparison to the generated random model pool (solid gray bars), the selected ensemble has a bimodal distribution (Fig. 6E). There is a major peak with Rg values around 2.3 nm and a minor peak around 3.5 nm. This is compatible with the coexistence in solution of compact and extended conformations, respectively. This is clearly observed in the representative structures selected by EOM (Fig. 6F).

### Analyzing the role of the CTD-CTD contacts in IB formation

**Fig 7:**
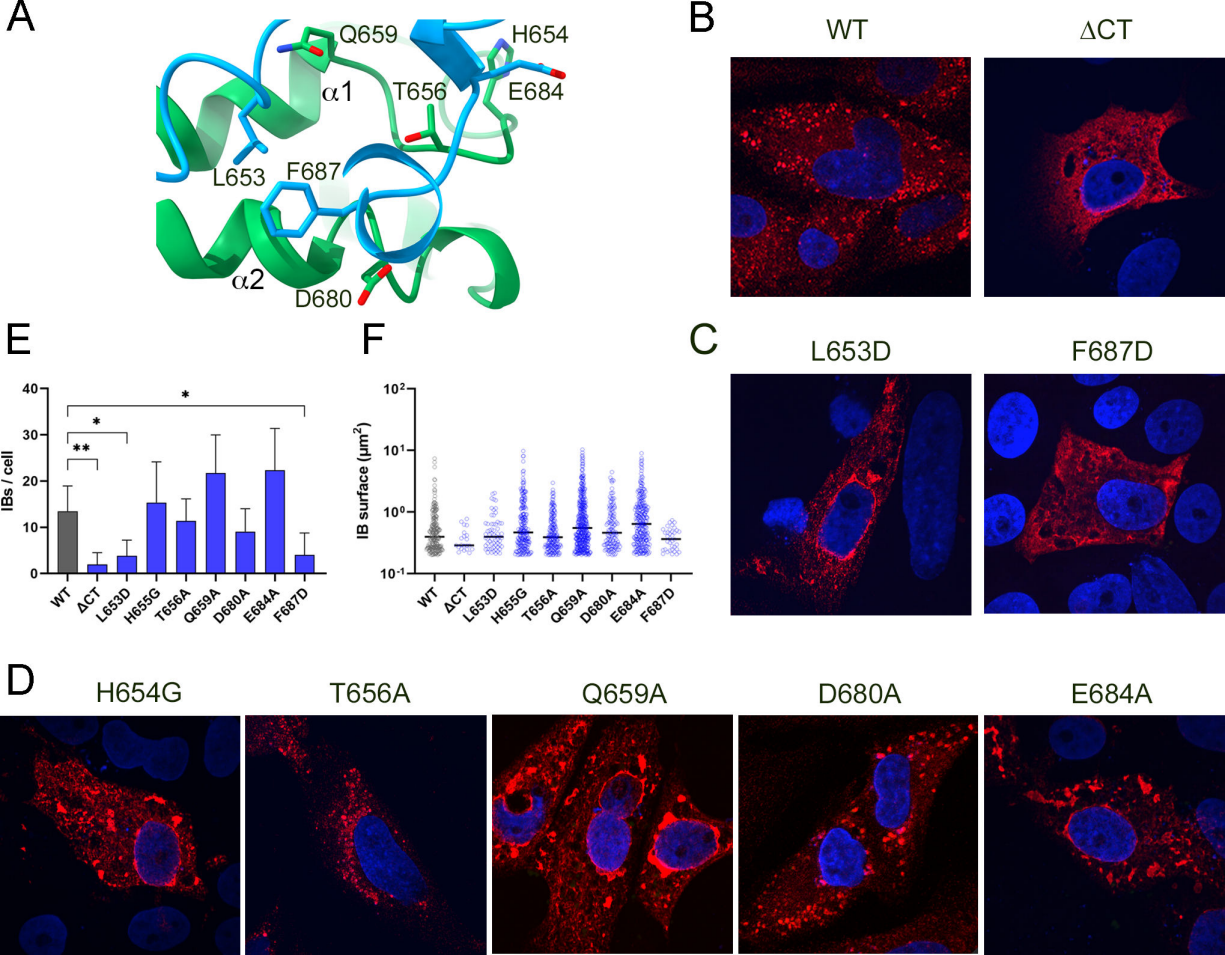
The MLAV NP CTD hydrophobic residues involved in domain oligomerization are required for IB formation. (**A**) The MLAV NP tail inter-protomer interactions, stabilizing the long CTD helicoidal oligomers. Contacts combine both polar and hydrophobic interactions. (**B**) IB formation in Vero cells transfected with wild-type MLAV NP full length, positive control (left panel). The MLAV NP_1–572_ construct, lacking CTD, was distributed sparsely throughout the cytoplasm, negative control (right panel). (**C**) Effect of the MLAV NP mutants, interfering with IB formation L653D (left panel) and F687D (right panel) (**D**) The MLAV NP mutants T656A, H654G, Q659A, D680A, and E684A do not essentially affect IB formation. (**E**) Quantification of the number of IBs per cell. IBs formed by the expression of wild-type NP and the different mutants were quantified, using the image-processing macro AggreCount (45), setting all the required parameters for intensity threshold and cell segmentation for all images, and excluding the perinuclear region (25 px) in the analyses. Only IBs with a surface >0.2 µm^2^ were considered. The number of IBs per image was divided by the number of transfected cells on each image. Means and standard deviations are shown. Asterisks indicate *P* values from one-way ANOVA for the differences compared to NP wild type (**, *P* < 0.01, *, *P* < 0.1), and the other comparisons were non-significant and are not shown in the graph. (**F**) Quantification of IB sizes in wild-type NP and in the different mutants. IBs were quantified as described in (E). The *y* axis is representedin the *y* axis is represented in logarithmic scale.

The essential role of the NP CTD in the formation of IBs has been previously demonstrated (15). Given that the three crystal structures solved in this work involved the presence of a multimeric helical assembly of this NP domain, we hypothesized that the interacting surfaces observed in our crystal structures (Fig. 3C) could be involved in crucial contacts leading to IB formation. To test this hypothesis, several independent point mutations were performed in the CTD of the full-length MLAV NP (Fig. 7A). Plasmids encoding the different NP mutants were transfected in Vero E6 cells and protein expression was monitored by indirect immunofluorescence and confocal microscopy. After 48 h post transfection (hpt), we clearly identified the presence of IBs in WT MLAV NP transfections, with size and morphology compatible with the IBs previously reported in other filoviruses (15, 19, 44) (Fig. 7B, left panel). As expected, the NP_1–572_ construct lacking CTD, was distributed sparsely throughout the cytoplasm and the formation of inclusion bodies was practically null (Fig. 7B, right panel). In addition, a markedly diffuse distribution of NP, comparable to that of the NP_1–572_ construct, was observed when the hydrophobic patch of surface II was disturbed by the substitutions F687→D and to a lesser extent L653→D, in the full-length protein (Fig. 7C). In contrast, point mutations in hydrophilic residues of surface I (T656A, H654G, Q659A, and D680A) and one in surface II (E684A) did not show significant differences in the formation of IBs, compared to the wild-type protein (Fig. 7D). The quantification of IB number in several images confirmed that the presence of IBs significantly decreased in mutants F687D and L653D (Fig. 7E and F). Curiously, the expression of the NP Q659A and H654G mutants showed some perinuclear accumulation of IBs which was also seen to a lesser extent in mutants L653D and E684A (Fig. 7C and D).

## DISCUSSION

MLAV is the unique member of the new genus Dianlovirus, within the *Filoviridae* family (8), and is closely related to MARV. MLAV represents a potential risk for human health since its viral replication machinery is functional in human-derived cell cultures (8). The NP and the RdRP L, with the cofactors VP35 and VP30, are the essential proteins of the replicative complex in all filoviruses. NP works like a scaffold for the replication machinery and protects the viral RNA genome. Its C-terminal domain protrudes from the viral nucleocapsid and is related to additional NP functions, including viral protein recruitment and host-protein interactions (27, 46, 47). The NP CTD is also an important antigenic determinant that could be used for virus identification (revised in reference 27). The structures of the NP CTD solved for different ebolavirus species show similar conformations between them (34, 36, 38, 39), although they present important differences when compared with the conformation of MARV NP CTD (37). Until now, neither the structure of MLAV NP, nor its N- or C-terminal domains was known. In this study, we determined the first X-ray structures of the MLAV NP CTD in three different crystal forms, showing important resemblances to the equivalent domain in MARV (Fig. 5A). The CTD structures of MLAV and MARV lack the two N-terminal helices that are characteristic of the CTDs from ebolavirus species (Fig. 5A). SAXS data also confirm the mixed flexible conformation of the MLAV NP_573–697_ domain in solution (Fig. 6).

Another peculiarity of the MLAV NP CTD is its quaternary organization, featured by a screw-like oligomer with a pseudo-threefold helical symmetry that is conserved in the AUs of the three crystals analyzed (Fig. 3A, 3B, 4A and 4B). The particular disposition of the CTD molecules in these helical oligomers, leaving the N-terminal ends of the domain exposed to the solvent (Fig. 3B), is perfectly compatible with the binding of the domain to the rest of the protein, in the context of the full-length NP. The packing in the three crystal forms analyzed, was built by interactions involving the full helical oligomers that change their relative disposition, originating the different space groups (Fig. 3A, 4A and B). In most cases, the visible CTD N-terminus does not participate in packing contacts, being exposed to the solvent. The absence of packing interactions in the N-terminal hairpin of the NP CTD structure has favored the small conformational differences seen in this region (Fig. 2D) that had not been observed in other NP CTD structures reported previously (34, 36 – 39). The only exception was found in a limited number of CTD molecules, within the helical oligomer of the P3_2_ crystals (Fig. 4B and D), where the CTD N-terminus appeared in close contact with other CTD molecules of neighboring helical oligomers in the crystal packing (Fig. 4D). In this position, the CTD N-terminal region extends from the β-hairpin in an orientation similar to the ebolavirus N-terminal α-helices (Fig. 5A), although in MLAV this region shows few elements of secondary structure (Fig. 4C and D).

Repetitive contacts of MLAV NP CTD forming the screw-like oligomer deserve special attention. They combine hydrophobic and polar residues in the interacting surfaces I and II (Fig. 3C). Neither the NP CTD-CTD contacts nor the presence of these helicoidal oligomers had been previously reported. However, the conservation of this quaternary organization in three different types of crystals, grown under different conditions, suggests that these oligomers are stable arrangements that might have a biological relevance in the context of a viral infection. A characteristic of filovirus-infected cells is the presence of IBs, formed in the host cell that accumulate viral as well as cellular proteins to enable efficient viral replication (19, 48). Recent studies revealed that these membrane-less virus-induced compartments have high structural and functional dynamics, presenting properties of liquid organelles (47), reviewed in reference (49). It is well characterized in EBOV and MARV, where the transient expression of NP alone is sufficient to induce IB formation in transfected cells (15, 50, 51). However, the absence of the NP CTD prevents IB formation, highlighting the crucial role of this region in the formation of these structures (15). The same authors also showed that the co-expression of VP35 surpassed the defect of NP CTD in triggering IB formation (15).

To confirm the involvement of MLAV NP CTD oligomerization in the formation of IBs, we generated mutants of the main interacting residues in the contact surfaces I and II and tested their effect on the formation of IBs in transfected cells (Fig. 7). As expected, the expression of MLVA NP full length in VERO E6 cells induced the formation of IBs (Fig. 7B, left panel), of size and morphology compatible with the IBs previously described in other Filoviruses (15, 50, 51). In contrast, point mutations in residues F687 or L653, disrupting hydrophobic surface II led to a diffuse cytoplasmic protein distribution, more evident in the F687D mutant (Fig. 7C). The diffuse protein distribution seen in the F687D mutant, closely resembled what was observed when the NP_1–572_ construct, lacking the 125 C-terminal residues, was expressed (Fig. 7B, right panel). These results are in agreement with the previously reported changes for other NPs within the *Filoviridae* family, after CTD removal (15). Interestingly, the structural superimposition of the NP CTD domains of MLAV and MARV shows that these two hydrophobic residues are conserved in both viruses (amino acids L651 and F685 in the MARV NP sequence; Fig. 5B), suggesting that this behavior could also be observed in MARV. The low similarity of the NP CTD structures between MLAV and ebolaviruses makes difficult the structural comparisons. However, superimpositions of the domain C-terminal helices show the presence of a phenylalanine side chain in ebolavirus CTDs (F662 in SEBOV), in an equivalent position to F687 in MLAV (Fig. 3C).

It has recently been shown that in addition to the CTD, the N- and central domains of EBOV NP could also control IB formation (15). However, the precise control mechanisms are not fully understood. Our results have contributed to increase the global picture of filovirus NP structures and pointed out the importance of NP CTD oligomerization as a control mechanism in the initial stages of IB formation, adding a new piece to the puzzle of IB biogenesis.

## MATERIALS AND METHODS

### Cloning of MLAV NP constructs

cDNA of MLAV NP was kindly provided by Drs. Xing-Lou Yang and Shi Zhengli (Wuhan Institute of Virology, Chinese Academy of Sciences) in the pCAGGS vector (4). For protein expression in *Escherichia coli*, the DNA sequence encoding NP CTD constructs NP_563–697_, NP_573–697_, and NP_600–697_ were amplified by PCR by using Phusion DNA polymerase (Thermo Fisher) according to the manufacturer’s instructions and purchased oligos (Table 3) flanked for BamHI and XhoI restriction sites with three extra nucleotides for proper restriction site function. The PCR fragments were purified, digested, and cloned into pGEX-6-P1 vector previously digested with identical enzymes. The obtained constructs were sequenced to verify the absence of mutations.

**TABLE 3:** Oligonucleotides employed for PCRs

Construct	Sequence (5′→3′)	Restriction site
NP_563–697_	CTCGGATCCCCTTTGTATGACACATCTACAAGTG	BamHI
	CGCCTCGAGTTAAAGGTTAAAAGCAACATTCTTC	XhoI
NP_573–697_	CTCGGATCCGGTGACATGGGAGCTGCAGAAC	BamHI
	CGCCTCGAGTTAAAGGTTAAAAGCAACATTCTTC	XhoI
NP_600–697_	CTCGGATCCGATACAGCCACAGTGCCATCAGCTCCTC	BamHI
	CGCCTCGAGTTAAAGGTTAAAAGCAACATTCTTC	XhoI
NP_L653D_	CCCAACTGACCTAGATCATAGAACTCCGCC	
	AACATAAATTCTCGGCCTTTGTTCGTCTTG	
NP_H654G_	CTGACCTACTTGCTAGAACTCCGCC	
	TTGGGAACATAAATTCTCGGCCTTTGTTC	
NP_T656A_	CTACTTCATAGAGCTCCGCCACAG	
	GTCAGTTGGGAACATAAATTCTCGG	
NP_Q659A_	CAGCGGTTCTTCTTGATGCTTTAGTCAATGAATATGAAAG	
	GCGGAGTTCTATGAAGTAGGTCAGTTGGGAACATAAATTC	
NP_D680A_	GAGTTGTCAGCGGACTGGCCTGAGATGACATTTG	
	GGTAGCGGAGAGAGGACTTTCATATTCATTGAC	
NP_E684A_	GTCAGATGACTGGCCTGCGATGACATTTGAAG	
	AACTCGGTAGCGGAGAGAGGACTTTC	
NP_F687Q_	CCTGAGATGACACAGGAAGAAAGGAAGAATGTTGC	
	CCAGTCATCTGACAACTCGGTAGCGGAGAG	
NP_His_tag_	TAAGCTAGCATCGAGGGCCGGGGC	
	CGCGCTAGCCTAGTGGTGATGATGGTGATGAAGGTTAAAAGCAACATTCTTCCTTTCTTC	
NP_ΔCT_	TCAAATTTCACTTGTAGATGTGTCATACAAAG	
	TAAGCTAGCATCGAGGGCCGGGGC	

Point mutations and C-terminal deletion in the full-length NP construct were introduced by PCR according to the previously described protocol (52), with the oligonucleotides shown in Table 3 for each point mutation. Briefly, the ORF cloned in the pCAGGS vector was amplified by PCR using Phusion DNA polymerase and introducing the point mutation in the corresponding Fw primer. The PCR products were treated with DpnI enzyme (Thermo Fisher) to eliminate the template DNA and then, visualized and purified from agarose gel by the NucleoSpin commercial kit, following the manufacturer’s instructions (Macherey-Nagel). The purified DNAs were phosphorylated with T4 polynucleotide kinase (New England Biolabs) and ligated with T4 DNA ligase after heat-shock transformation in *E. coli* DH5 alpha. After verification of the point mutations by sequencing, a 6xHis tag was added to the C-terminal end of NP, using the same PCR-based method (52) and introducing the coding sequence for the His tag in the Rv primer (Table 3).

### Protein expression and purification

NP CTD constructs cloned in the pGEX-6-P1 vector (NP_563–697_, NP_573–697_, and NP_600–697_) were transformed into *E. coli* BL21 (DE3) cells by heat shock method. After growth of the bacterial cultures in LB medium at 37°C with 50 µg/mL ampicillin until OD_600_ = 0.6, the expression of the recombinant proteins was induced by the addition of 0.5 mM IPTG, overnight at 22°C. Cell cultures were harvested by centrifugation (4,000 g, 30 min, JLA-9.1000 rotor), the cellular pellets were washed with phosphate-buffered saline (PBS) and stored at −20°C.

For protein purification, each bacterial pellet was resuspended in 50 mL lysis buffer [50 mM Tris pH 8.0, 300 mM NaCl, 5% (vol/vol) glycerol, 1 mM DTT, 5 mM MgCl_2_, 1 mM DNase, 1 mM RNase, 100 µg/mL lysozyme, and one EDTA-free complete protease inhibitor pill] and disrupted in a French press (1.35 kbar) at 4°C. The lysate was clarified by centrifugation at 19,000 rpm for 30 min in a JA 25.50 rotor and filtrated with 0.22 µm syringe filters. Clarified lysates were loaded into a 5-mL GST-trap HP column (GE Life Sciences) at 0.2 mL/min and washed with 10 column volumes (CVs) of wash buffer (50 mM Tris pH 8.0, 300 mM NaCl, and 1 mM DTT) at 0.5 mL/min. The fusion protein was digested in column with the addition of 1 CV of digestion buffer [50 mM Tris pH 8.0, 300 mM NaCl, 5% (vol/vol) glycerol, 1 mM DTT] and 10U of PreScission protease, ON at 20°C. The recombinant proteins of interest were recovered by washing the column with 4 CVs of buffer (50 mM Tris pH 7.0, 500 mM NaCl, 5 mM DTT, 5% (vol/vol) glycerol), and GST eluted with 4 CVs of 10 mM reduced glutathione in the washing buffer. The NP CTD constructs were concentrated to 10 mg/mL, using Amicon centrifugal filters (3 kDa MWCO) and subjected to size-exclusion chromatography (SEC) purification on a Superdex 200 increase 10/300 column (GE Healthcare) and buffer 50 mM Tris pH 8.0, 500 mM NaCl, 5 mM DTT, and 5% (vol/vol) glycerol. Fractions containing NP CTD proteins were pooled and concentrated to 20 mg/mL using Amicon filters (3 kDa MWCO).

### Crystallization of NP CTD

Initial crystallization trials of MLAV NP CTD constructs were performed by the sitting-drop vapor diffusion method at 293K in 96-well Greiner plates using a nanoliter-drop dispenser robot Phoenix (Art Robbins Instruments, Inc.) and multiple crystallization screens available at the Automated Crystallography Platform (PAC) (IBMB-CSIC, Barcelona). Protein solution (20 mg/mL in 50 mM Tris pH 8.0, 500 mM NaCl, 5 mM DTT, 5% (vol/vol) glycerol) droplets of 100 nL were mixed with 100 nL of precipitant solution and the volume reservoir was 100 µL. Small crystals appeared within 2 weeks in several conditions for NP_573–697_ construct: (i) 2 M ammonium sulfate, 0.1 M Bis-Tris pH 5.5; (ii) 1 M Tri-sodium citrate dihydrate, 0.1 M cacodylate pH 6.5; (iii) 0.2 M NaCl, 0.1 M cacodylate pH6.5, 2 M ammonium sulfate; (iv) 0.05 M di-potassium hydrogen phosphate, 20% (wt/vol) PEG 8000; (v) 0.2 M magnesium acetate tetrahydrate, 0.1 M cacodylate pH 6.5, 20% (wt/vol) PEG 8000; (vi) 0.2 M ammonium acetate, 0.1 M tri-sodium citrate pH 5.6, 30% (wt/vol) PEG 4000; and (vii) 0.2 M ammonium sulfate, 0.1 M MES pH 6.5, 30% (wt/vol) PEG 5000 MME. Also for NP_600–697_ construct, small crystals appeared after 1 week in: (i) 0.05 M di-potassium hydrogen phosphate, 20% (wt/vol) PEG 8000; (ii) 0.2 M magnesium acetate tetrahydrate, 0.1 M cacodylate pH 6.5, 20% (wt/vol) PEG 8000; (iii) 0.5 M ammonium sulfate, 0.1 M Tri-sodium citrate pH 5.6, 1 M lithium sulfate monohydrate; and (iv) 2 M ammonium sulfate.

After optimization of each condition in 96-well plate using 400 nL drops, the best crystals were harvested in cryo-loops (Molecular Dimensions), soaked for 1 min in a reservoir solution containing 20% (vol/vol) glycerol, and flash-frozen in liquid nitrogen.

The three crystals that diffracted at the highest resolution were obtained in 0.2 M ammonium sulfate, 0.1 M MES pH 6.5, 30% (wt/vol) PEG 5000 MME (P1 crystal); in 0.2 M NaCl, 0.1 M cacodylate pH 6.5, 2 M ammonium sulfate (P2_1_ crystal), and in 0.1 M NaCl, 0.1 M HEPES pH 7.0, 1.6 M ammonium sulfate (P3_2_ crystal).

### X-ray diffraction data collection and processing

Diffraction data were acquired at 100K using Synchrotron radiation at ALBA (XALOC beamline) and Diamond (I03 beamline). Data were processed using XDS (53). Space group determination and data reduction were performed with POINTLESS/AIMLESS (54). Inspection of the unmerged data using the STARANISO software (55), currently available in the web server (http://staraniso.globalphasing.org), were further employed anisotropic correction of all X-ray data to fit the ellipsoid diffraction cutoff, according to default criteria (Rpim = 0.6, *I*/σ*I* = 2.0 and CC1/2 = 0.3). STARANISO analyses the anisotropy decay of the mean intensities (*I*), constrained by the crystal symmetry, modifies the TRUNCATE procedure, and analyses the decay of the local average of *I*/σ(*I*) in different directions, providing the basis for an anisotropic resolution cutoff.

Applied anisotropy correction parameters and the best resolution limits are detailed in Table 1 for the different crystals. The P1 crystals diffracted at 3.6 Å on 0.959a*+0.284b*+0.043c*, 4.3 Å on −0.046a*+0.998b*+0.042c* and 3.2 Å on 0.100a*−0.001b*+0.995c* axis. The P3_2_ crystals diffracted at 4.1 Å on 0.894a*±0.447b*, 4.1 Å on b* and 7.0 Å on c* axis, while P2_1_ crystals diffracted at 3.4 Å on 0.920a*+0.392c*, 7.3 Å on b* and 5 Å on −0.094a*+0.996c* axis.

Solvent content was calculated using Matthews-coef (56, 57) software available in CCP4 suite (58).

The structures were solved by molecular replacement using PHASER (59) available at Phenix (60), using the coordinates of the NP C-terminal domain of MARV (PDB: 4W2Q, residues 633–695) as initial model. Solution models were manually rebuilt in COOT (61) and refined using Phenix refine (62) using secondary structure restraints and each chain as TLS groups. Composite omit map was also calculated using Phenix to help in model building. Data refinement statistics are listed in Table 1. Superimpositions of structures were carried out using Coot. Calculations of contact surfaces were performed with PISA (63). Figures were prepared with ChimeraX (64).

### Molecular weight analysis

SEC combined with light scattering analysis was used to analyze the molecular weights of the different protein constructs and the sample quality after purification. The SEC data were measured using a Superdex 200 10/300 Gl column (GE Healthcare; MW range: 10–600 kDa), connected to high-performance liquid chromatography system (Shimadzu), equipped with an autosampler. The elution from SEC was monitored by a UV detector, differential refractometer (OPTI-rEx, Wyatt Corp.) and static, multiangle laser LS detector (DAWN-HELEOS, Wyatt Corp.). The SEC-UV/MALS/RI system was equilibrated in 50 mM Tris pH 8.0, 500 mM NaCl, 5 mM DTT, and 5% (vol/vol) glycerol at the flow rate of 0.5 mL/min. The ASTRA 7 software (Wyatt Corp.) was used for data collection and analysis.

### Small-angle X-ray scattering

NP_573–697_ protein was prepared as described in the protein purification section and analyzed after two rounds of dialysis at 4°C over a period of 12 h against storage buffer [50 mM Tris pH8.0, 500 mM NaCl, 5% (vol/vol) glycerol, and 5 mM DTT]. An aliquot of post dialysis buffer was filtered (0.22 µm) and kept as the matched solvent blank for the SAXS measurements. The protein samples and buffer were filtered with centrifugal filter devices (0.22 µm, Durapore PVDF, Millipore) immediately before measurements. Protein concentration was evaluated spectrophotometrically at 280 nm using an extinction coefficient of 0.8457 (mg/mL)^−1^ cm^−1^.

SAXS data were recorded at the BM29 beamline (ESRF, Grenoble) with an X-ray source of 0.9678 Å wavelength and 2.81 m sample to detector distance (Pilatus3 2M). For continuous-flow batch mode measurements, a range of NP_573–697_ concentrations (50 µL sample, 1–4 mg/mL) were automatically loaded in a quartz glass capillary (1 mm diameter) and data were collected in a series of 10 consecutive frames, each one lasting 1 s, flanked by two exposures to buffer sample. All the experiments were performed at 20°C.

Data reduction was performed using Primus and the posterior analysis using the software from the ATSAS package (65). The radius of gyration (Rg) was calculated using the Guinier approximation (66) with data limit qmaxRg <1.3 where *q* = 4*π* sin *θ*/*λ*. Pair distance distribution function PI and the Dmax were calculated using the indirect Fourier transform method implemented in GNOM (67). Molecular weights were calculated using the volume correlation (Vc) method (40), implemented in ATSAS package (65).

Modeling of protein structure in solution was performed using the EOM, available in the ATSAS online web server (68). The monomeric crystal structure (this work) was employed as a rigid body model, and the flexible N-terminal region was modeled by ranch software, generating a pool of models (10,000), where a genetic algorithm selected an ensemble that fitted the data best.

### Cell culture, transfection, and indirect immunofluorescence analysis

VERO E6 cells were maintained in Dulbecco’s modified Eagle’s medium (Thermo Fisher Scientific), supplemented with 2.5 µg/mL amphotericin B, 100 U/mL penicillin, 100 µg/mL streptomycin (Thermo Scientific), and 10% fetal bovine serum (GIBCO). Cells were grown at 37°C and 5% CO2.

For intracellular visualization of MLAV NP WT and mutants, VERO E6 cells were placed on 12 mm coverslips and grown for 24 h before transfection. METAFECTENE PRO (Biontex) was employed as a reagent for transfection of 500 ng of pCAGGS, each vector encoding a C-terminal His-tagged version of the different NP constructs, following the manufacturer’s instructions. At 48 h post transfection, cells were fixed with 4% paraformaldehyde in PBS at room temperature (RT) for 15 min. Coverslips were subsequently incubated with 0.1% (vol/vol) Triton X-100 in PBS for 10 min and incubated, 10% FBS in PBS, for 1 h at RT for cell permeabilization and blocking, respectively. Proteins were labeled by incubating the coverslips with rabbit anti-His tag antibody, diluted 1:1,000 for 1 h at 37°C. After six washes with PBS, the coverslips were incubated with anti-Rb Fc, marked with Alexa594 and diluted 1:1,000. Both antibodies were diluted in 5% FBS/PBS. Nuclei were stained with DAPI (dilution 1:2,000) for 15 min and coverslips were mounted with ProLong Gold (Thermo Scientific). Images were visualized with the DragonFly 505 (ANDOR) microscope, with a 100× oil immersion objective. Confocal images were analyzed with the FIJI software (69). Each Fluorescence channel was deconvolved and denoised. The IBs were detected, using the image-processing macro AggreCount (45), setting all the required parameters for intensity threshold and cell segmentation for all images, and excluding the perinuclear region (25 px) in the analyses. Only IBs with a surface >0.2 µm^2^ were considered. Six images containing 20 cells were analyzed for each protein construct.

### Statistical analysis

One-way analysis of variance (ANOVA) with Dunnett’s multiple-comparison test (Fig. 7E) was performed using GraphPad Prism.

## Data Availability

The refined coordinates and structure factors are available at the Protein Data Bank (https://www.rcsb.org) under accession codes 8P10, 8P24, and 8P0Y for the NP CTD P1, P2_1_, and P3_2_ crystals, respectively.
